# Bile Salts Affect Expression of *Escherichia coli* O157:H7 Genes for Virulence and Iron Acquisition, and Promote Growth under Iron Limiting Conditions

**DOI:** 10.1371/journal.pone.0074647

**Published:** 2013-09-10

**Authors:** Steve Hamner, Kate McInnerney, Kerry Williamson, Michael J. Franklin, Timothy E. Ford

**Affiliations:** 1 Department of Microbiology, Montana State University, Bozeman, Montana, United States of America; 2 Department of Microbiology and Functional Genomics Core Facility, Montana State University, Bozeman, Montana, United States of America; 3 University of New England, Biddeford, Maine, United States of America; University of Hyderabad,, India

## Abstract

Bile salts exhibit potent antibacterial properties, acting as detergents to disrupt cell membranes and as DNA-damaging agents. Although bacteria inhabiting the intestinal tract are able to resist bile’s antimicrobial effects, relatively little is known about how bile influences virulence of enteric pathogens. *Escherichia coli* O157:H7 is an important pathogen of humans, capable of causing severe diarrhea and more serious sequelae. In this study, the transcriptome response of *E. coli* O157:H7 to bile was determined. Bile exposure induced significant changes in mRNA levels of genes related to virulence potential, including a reduction of mRNA for the 41 genes making up the locus of enterocyte effacement (LEE) pathogenicity island. Bile treatment had an unusual effect on mRNA levels for the entire flagella-chemotaxis regulon, resulting in two- to four-fold increases in mRNA levels for genes associated with the flagella hook-basal body structure, but a two-fold decrease for “late” flagella genes associated with the flagella filament, stator motor, and chemotaxis. Bile salts also caused increased mRNA levels for seventeen genes associated with iron scavenging and metabolism, and counteracted the inhibitory effect of the iron chelating agent 2,2’-dipyridyl on growth of *E. coli* O157:H7. These findings suggest that *E. coli* O157:H7 may use bile as an environmental signal to adapt to changing conditions associated with the small intestine, including adaptation to an iron-scarce environment.

## Introduction

Bacteria must resist the antimicrobial properties of bile in order to survive in the small intestine [1]. Bile salts act as a biological detergent that aids in digestion through emulsification of fatty foods. These detergent properties allow bile to disrupt the membranes of bacteria. Bile salts also act as DNA-damaging agents [2] and can cause oxidative stress [3]. Bacteria that have adapted to life in the intestine have developed mechanisms of resistance to bile, including modified membrane structures that reduce bile permeability, and efflux pumps that are able to transport bile out of the cell [1].

Bile has been proposed to be a signal to enteric pathogens of having entered the small intestine and for the regulated expression of virulence [4]. Bile is released into the intestine at the duodenum. Bile salt concentration generally varies from 0.2–2.0% in the small intestine, although dietary intake and nourishment status can greatly affect bile levels [4]. The concentration decreases as bile salts are passively reabsorbed along the entire length of the small intestine and then actively reabsorbed in the ileum. This concentration gradient in the small intestine and the very low levels of bile salts remaining in the large intestine may have significant consequences for the temporal expression of virulence genes by pathogenic bacteria. Also, closely related bacteria may have developed different mechanisms of responding to bile [5]. How different pathogens respond to bile may influence where in the intestine they express virulence determinants.

Serotype O157:H7 belongs to a pathotype of diarrheagenic *E. coli* referred to as EHEC (for enterohemorrhagic *E. coli*), able to cause diarrhea, hemorrhagic colitis and the more severe complication of hemolytic uremic syndrome. The major virulence factors described for *E. coli* O157:H7 include the Shiga-like toxins 1 and 2 [6,7], and an adhesin, intimin, and associated factors encoded by the locus of enterocyte effacement (LEE) pathogenicity island [8,9]. Shiga toxins bind to specific host cells receptors and, once internalized by infected host cells, cause cell death by inhibiting protein synthesis. The 41 genes and open reading frames of the LEE code for intimin, the translocated intimin receptor, and a type III secretion system that introduces protein effectors into host cells [8,9]. EHEC bacteria expressing the LEE genes and colonizing the intestine cause an attaching-and-effacing pathogenesis characterized by: intimate adherence to host cells, disruption of target cell cytoskeleton elements and creation of actin pedestals within the epithelial cells that the bacteria are attached to, and reduced integrity of tight junctions causing loss of intestinal epithelial barrier function [8,9].

The LEE pathogenicity island is also found in another related pathotype known as EPEC (for enteropathogenic *E. coli*). Although the genetic elements of the LEE pathogenicity islands of EHEC and EPEC share many similarities [8], there are key differences between the two LEE systems that may account for the different forms of disease manifested by the two pathotypes - while EPEC infects the small intestine, EHEC O157:H7 manifests disease primarily in the large intestine. For EPEC to express the attaching-and-effacing phenotype in the small intestine, and for EHEC to express disease in the large intestine, expression of the LEE-encoded proteins is necessary at different stages of passage through the intestine for these two different pathotypes. Regulation of LEE expression in EHEC differs from that in EPEC.

Recent studies have described genetic and phenotypic differences expressed by various strains of O157:H7 [10–13], and have begun to identify gene products and pathways linked to early stage colonization and adhesion preceding the intimate attachment to intestinal epithelium associated with intimin/LEE [14–19]. One study has also employed microarray analysis to examine how bile stress affects the expression of virulence genes and bile resistance strategies in *E. coli* O157:H7 [20]. In this study, we describe an O157:H7 transcriptome associated with bile and how bile may be affecting the virulence properties of the bacteria prior to intestinal colonization. Because bile is introduced into the duodenum, a site of active iron absorption and creation of an iron-scarce environment, we also examine the growth of bile-treated O157:H7 cells related to an iron stress response.

## Materials and Methods

### Bacterial strain used - background

The strain of O157:H7 used in these experiments (DEC 3A, original strain number 3299-85) was isolated from a human case of hemorrhagic colitis in 1985 (STEC Center; http://www.shigatox.net) and was obtained from R. Wilson, *Escherichia coli* Reference Center, Pennsylvania State University [21]. Complete genome sequences have been obtained for the Sakai and EDL933 strains of O157:H7 and incorporated in the design of the commercially produced microarray chips (Affymetrix) used in the study described herein. The *eae* (intimin) gene of DEC 3A has been sequenced [22]; BLAST analysis shows 100% identity with the *eae* nucleotide sequences for both the Sakai and EDL933 strains of O157:H7. The nucleotide sequence of the *fliC* (flagellin) gene of DEC 3A has also been determined [23]; BLAST comparison with the *fliC* sequence of the Sakai strain of O157:H7 shows identify for 1679/1680 base pairs, and comparison with *fliC* of the EDL933 strain of O157:H7 shows identify for 1678/1680 base pairs. The long polar fimbriae (lpf) operon of DEC 3A has recently been sequenced and found to share 100% identity with the Sakai strain sequence (M. Maddaloni, personal communication).

### Bacterial growth under aerobic conditions

Batch cultures of planktonic bacteria exposed to atmospheric air (aerobic conditions) were incubated with shaking in LB broth (Luria-Bertani / Miller broth, Difco) with and without the presence of 0.8% Bacto bile salts (Difco 0129-02-4). Triplicate cultures of both control and bile-treated cells were grown at 37°C and harvested during logarithmic phase growth. Iron concentration of fresh and spent medium (with and without bile added) was measured using an Iron Test Kit (Hach). Each of the six cultures (triplicate cultures for each treatment group, control and bile-treated cells), was processed independently with respect to growth, RNA extraction/preparation, and microarray hybridization.

### Measurement of bacterial growth

The effects of bile salts and iron depletion on cell growth were examined by growing O157:H7 cells under four medium treatments: 1) LB broth alone; 2) LB broth containing 0.8% bile salts; 3) LB broth containing 250 µM of the iron chelating agent 2,2’-dipyridyl (DPD) (Fluka); and 4) LB broth containing both bile salts and DPD. Cells grown overnight on LB agar at 37°C were inoculated into 10ml of LB broth and incubated with shaking at 37°C for four hours. Aliquots of the broth culture were diluted 1:100 into each of the four LB media (with and without bile salts/DPD). The diluted cells were mixed thoroughly by gentle vortexing followed by incubation with shaking for five minutes at 37°C, and each of the four media with cells were dispensed in triplicate, 200 µl per well, into 96 well plates. Growth was measured using a Synergy2 Multi-Mode Microplate Reader (BioTek Instruments, Inc). The 96 well plate was incubated at 37°C with shaking (medium speed setting) in the Synergy2 microplate reader, and growth monitored by recording OD_600_ every 15 minutes for a total of 20 hours. Four independent experiments were conducted. Doubling times during logarithmic growth phase were calculated using exponential regression analysis with Doubling Time software (http://www.doubling-time.com/compute.php). Doubling time calculations were made using data values for the time interval between 30 to 90 minutes (inclusive) when exponential growth curves were steepest.

### RNA extraction and cleanup

Cells were collected from each of the six cultures (three of control and three of bile treated) by centrifugation and each culture processed individually. RNA was isolated using hot phenol extraction. Briefly, pelleted cells were suspended in 1.5 mls lysis buffer (0.15 M sucrose, 0.01 M sodium acetate, pH 4.5) and 1.5 mls 2% sodium dodecyl sulfate. Following the addition of 3 mls of water-saturated phenol, the mixture was incubated for 5 minutes at 65°C with frequent vortexing. Next, 3 mls of chloroform were added and the mixture was centrifuged for 30 minutes at 4°C to allow for phase separation. The aqueous layer was removed by pipette, precipitated overnight, washed with 70% ethanol, and then resuspended in RNase-free water. Next, the RNA was cleaned on an RNeasy column (Qiagen) following the manufacturer’s mini cleanup protocol. The RNA was then subjected to two 30 minute Turbo DNase (Ambion) treatments, followed by inactivation of the reagent and overnight precipitation to further clean and concentrate the RNA. RNA concentrations were measured using a Nanodrop spectrophotometer, and RNA quality assessed with the Agilent Bioanalyzer 2100. All six RNA samples (three for each treatment group of control vs. bile) were confirmed as having RNA integrity numbers of 9.6-10.0 before use in subsequent applications.

### cDNA synthesis and preparation

Each of the six RNA samples was reverse transcribed at 42°C overnight. Each reaction included 1 µl of Poly-A control RNA (Affymetrix), 1 µl random primers (Invitrogen), 1 µl Superase-In (Ambion), 6 µl 5x first strand buffer (Invitrogen), 3 µl 0.1 M DTT, 0.6 µl 100mM dNTPs (Invitrogen), 3 µl Superscript II (Invitrogen), and 8 µg RNA. After synthesis, the remaining RNA was hydrolyzed by the addition of 0.5 M EDTA (Invitrogen) and 1 N sodium hydroxide. Next, the cDNA samples were neutralized with 1 N HCl and 1 M sodium acetate. The reactions were then cleaned on MinElute columns (Qiagen) according to the manufacturer’s instructions. The resulting cDNA was fragmented with 0.6 Units DNase I (GE Healthcare) per microgram cDNA for 10 minutes at 37°C. The enzyme was inactivated by heating to 98°C for 10 minutes. The resulting cDNA fragment sizes, assessed using an Agilent RNA 6000 nano assay, ranged from 35 to 200 base pairs. Fragmented cDNA was terminally labeled by incubation with 10 µl 5x reaction buffer, 2 µl GeneChip DNA labeling reagent (Affymetrix), and 2 µl terminal deoxynucleotidyl transferase (Promega) for 60 minutes at 37°C.

### Hybridization, scanning and analysis

Labeled cDNA was hybridized overnight at 45°C with constant rotation to Affymetrix *E. coli* Genome 2.0 GeneChip microarray chips. The Genome 2.0 microarray reflects genic and intergenic sequences for the two sequenced O157:H7 strains, EDL933 and Sakai, as well as a uropathogenic strain and laboratory strain K12 (Affymetrix). Microarrays were washed and stained using a GCOS Fluidics Station 450 and scanned with an Affymetrix 7G scanner. Affymetrix GCOS v 1.4 was used to generate CEL and CHP files. CEL files were imported into FlexArray v 1.4.1 [24] for QC and gc-RMA normalization [25]. The data were filtered for baseline signal intensity and modest fold change, and a cyber-T test [26] was used to identify genes with statistically significant changes in expression. Genesis 1.7.5 [27] was used to generate hierarchical trees.

### Quantitative reverse transcription PCR (qRT-PCR)

qRT-PCR was performed for selected genes to confirm microarray results. The same RNA preparations (triplicate sets for each treatment group) used for DNA microarray analysis were also used for qRT-PCR. Primer pair sequences for LEE genes were designed using the Frodo Primer3 program [28]; sequences for *marA*, *acrA*, *ompF*, *tdcA*, and *gapdh* were adopted from a previous study [29]. *gapdh* was selected as the housekeeping gene reference. Real time qRT-PCR was performed with a Corbett Rotor Gene-3000 and a Quantitect SYBR, Green RT-PCR kit (Qiagen) following manufacturer’s guidelines; 25 µl reaction volumes were used, with each reaction including 20ng of RNA.

### Soft agar motility assay

Soft agar plates were prepared using LB supplemented with 0.3% agar (Difco). O157:H7 bacteria were grown in LB broth with and without 0.8% bile salts through mid-log phase. 5 µl volumes of each treatment group were then spotted in triplicate on soft agar plates and incubated at 37°C with periodic observation and measurement of growth circles for up to 24 hours. This assay was also performed with either 0.8% or 0.1% bile salts being incorporated into the soft agar medium.

### Microarray data

The microarray data set presented in this publication has been deposited in the Gene Expression Omnibus and is accessible through GEO Series accession number GSE20380 (http://www.ncbi.nlm.nih.gov/geo/query/acc.cgi?acc=GSE20380). 

## Results

### Bile causes reduced transcription of the LEE pathogenicity island virulence determinants in *E. coli O157:H7*

For transcriptome analysis, a human-associated strain of O157:H7 was grown for three hours (to mid-log phase) in the presence or absence of 0.8% bile salts. In a study of normal, healthy females and males, the mean transit time for food/digestive products to pass through the small intestine was measured to be in the range of 179 to 190 minutes, or about three hours [30]. Accordingly, O157:H7 cells were incubated in LB medium containing bile salts for three hours, since bacteria mixed with digestive products are exposed to bile during transit through the small intestine. While the concentration of bile varies with the individual, 0.8% is in the range of an average concentration that would be found *in vivo*. The pH of the LB medium, with and without bile, was 7.0 before culture and after completion of culture. The experiments were carried out under aerated conditions. The intestine has historically been considered to be an anaerobic environment. However, the intestine actually harbors constantly changing microenvironments with respect to oxygen concentration, varying from aerobic to microaerobic, due to the contribution of oxygen from ingested or swallowed air, and diffusion of oxygen from richly oxygenated intestinal epithelial tissue [31–34]. In one study, it was concluded that aerobic respiration was essential for successful colonization of the mouse intestine by both commensal and pathogenic *E. coli* [30]. Accordingly, *E. coli* O157:H7 was grown in batch culture with exposure to air in these studies.

Shiga toxin 1 and 2 (*stx1/2*) mRNA levels remained unchanged in the presence or absence of bile (Table 1). However, mRNA levels for 38 of the 41 genes associated with the Type III secretion system of the LEE pathogenicity island were reduced two- to six-fold with bile treatment compared to the untreated controls (Table 2). To confirm the effect of bile on LEE genes, mRNA levels for one gene from each of the four operons of the LEE pathogenicity island that had statistically significant, reduced mRNA levels were measured using qRT-PCR (Table 3). The qRT-PCR data are consistent with the microarray data and support the apparent downregulation of LEE genes by bile addition.

**Table 1:** Lack of change in mRNA levels for Shiga-like toxin genes in O157:H7 cells treated with 0.8% bile salts relative to control (calculations of “fold change” are based on triplicate culture/microarray experiments for each treatment).

**Gene symbol**	**Gene name & function**	**Fold change**	**p-value**
*stx1A*	Shiga-like toxin 1 - virulence	1.06 (insignificant)	NS
*stx1B*	"	1.15 (insignificant)	NS
*stx2A*	Shiga-like toxin 2 - virulence	1.06 (insignificant)	NS
*stx2B*	"	.90 (insignificant)	NS

“NS” denotes “not significant” for p-values > 0.05.

**Table 2:** Downregulation of Locus of Enterocyte Effacement (LEE) mRNAs in O157:H7 cells treated with 0.8% bile salts relative to control.

**Locus Tag**	**Gene Symbol**	**Fold change**	**p-value**
Z5100	*espF*	.36	.002
Z5102	-	.38	.009
Z5103	*escF*	.30	< .001
Z5104	*cesD2*	.38	.003
Z5105	*espB*	.62	NS
Z5106	*espD*	.56	NS
Z5107	*espA*	.57	NS
Z5108	*sepL*	.38	< .001
Z5109	*escD*	.31	< .001
Z5110	*eae*	.43	.025
Z5111	*cesT*	.38	.003
Z5112	*tir*	.45	< .001
Z5113	*map*	.31	< .001
Z5114	*cesF*	.27	< .001
Z5115	*espH*	.44	.002
Z5116	*sepQ*	.19	< .001
Z5117	-	.25	< .001
Z5118	-	.29	< .001
Z5119	*escN*	.26	< .001
Z5120	*escV*	.34	< .001
Z5121	-	.30	< .001
Z5122	*sepZ*	.30	< .001
Z5123	-	.18	< .001
Z5124	*escJ*	.16	< .001
Z5125	*sepD*	.22	< .001
Z5126	*escC*	.20	< .001
Z5127	*cesD*	.14	< .001
Z5128	*grlA*	.28	< .001
Z5129	*grlR*	.33	< .001
Z5131	-	.50	< .001
Z5132	*escU*	.15	< .001
Z5133	*escT*	.15	< .001
Z5134	*escS*	.26	< .001
Z5135	*escR*	.26	< .001
Z5136	-	.17	< .001
Z5137	-	.26	< .001
Z5138	*cesAB*	.27	< .001
Z5139	-	.33	< .001
Z5140	*ler*	.39	< .001
Z5142	*espG*	.47	< .001
Z5143	-	.55	.007

Calculations of “fold change” are based on triplicate culture/microarray experiments for each treatment.

“NS” denotes “not significant” for p-values > 0.05.

**Table 3:** Changes in mRNA levels measured using quantitative RT-PCR vs. microarray in O157:H7 cells treated with 0.8% bile salts relative to control.

**Gene Symbol**	**Fold change (quantitative RT-PCR** )	**Fold change (miroarray data**)
*eae* (LEE)	.56	.43
*escN* (LEE)	.50	.26
*escC* (LEE)	.42	.20
*ler* (LEE)	.52	.39
*ompF*	.12	.14
*marA*	2.08	2.01
*acrA*	2.00	1.89
*tdcA*	.51	.30

Calculations of “fold change” are based on triplicate culture/microarray experiments for each treatment. The same RNA preparations (three independent preparations for each treatment) were used for RT-PCR as were used for the microarrays.

### Bile influences mRNA levels for flagellar and chemotaxis components

Bile induced an unusual effect on the mRNA levels of flagella and chemotaxis genes. The mRNA levels for regulatory genes/operons (*flhDC*, *flgMN*, and *fliAZY*) controlling flagellar biosynthesis remained relatively unchanged under bile treatment compared to untreated controls (Table 4). However, all of the genes encoding components of the basal body-hook structure (termed middle genes) had two- to four-fold increased mRNA levels in the bile treated cells (Table 4). In contrast to the basal body-related genes, all of the late flagellar genes, including the genes encoding elements of the flagellar filament and motor stator had approximately two-fold reductions in mRNA levels (Table 5). The co-regulated chemotaxis signal transduction system also showed reduced mRNA levels due to the addition of bile (Table 5).

**Table 4:** Upregulation of mRNA levels for Class 2, or “middle” flagellar genes encoding hook-basal body structures in O157:H7 cells treated with 0.8% bile salts relative to control.

**Gene**	**Protein function**	**Fold change**	**p-value**
Class 1 (early) genes – *flhDC* operon			
*flhD*	transcriptional activator-flagellar class 2 operons	0.84	NS
*flhC*	transcriptional activator-flagellar class 2 operons	0.83	NS
Class 2 (middle) genes – *fliLMNOPQR* operon			
*fliL*	flagellar basal body-associated protein	2.53	.006
*fliM*	flagellar motor switching & energizing	3.22	.004
*fliN*	flagellar switch protein	3.48	.004
*fliO*	flagellar biosynthesis protein	3.46	.007
*fliP*	flagellar biosynthesis protein	3.54	.010
*fliQ*	flagellar biosynthesis protein	3.91	.010
*fliR*	flagellar biosynthesis; export pore protein	2.99	.031
Class 2 (middle) genes – *fliE/fliFGHIJK* operon			
*fliE*	flagellar hook-basal body protein	2.62	.009
*fliF*	flagellar basal body MS-ring and collar protein	3.12	.004
*fliG*	flagellar motor switch protein	4.79	.002
*fliH*	flagellar biosynthesis protein	3.42	.002
*fliI*	flagellum-specific ATP synthase	3.27	.008
*fliJ*	flagellar biosynthesis chaperone	2.46	.024
*fliK*	flagellar hook-length control protein	2.57	.009
Class 2 (middle) genes – *flgAMN* operon; also containing class 3 genes - *flgMN*			
*flgA*	flagellar basal body P-ring biosynthesis protein	4.25	< .001
*flgM*	anti-FliA (anti-sigma 28) factor; FlhD regulator	0.89	NS
*flgN*	initiation of flagellar filament assembly	0.80	NS
Class 2 (middle) genes – *flgBCDEFGHIJ* operon			
*flgB*	flagellar basal body rod protein	4.47	< .001
*flgC*	flagellar basal body rod protein	2.78	< .001
*flgD*	flagellar hook assembly protein	3.46	.002
*flgE*	flagellar hook protein	3.07	.001
*flgF*	flagellar basal body rod protein	2.68	.002
*flgG*	flagellar basal body rod protein	2.64	.002
*flgH*	flagellar synthesis, basal body L ring lipoprotein	3.29	.004
*flgI*	flagellar basal body P-ring protein	1.94	.019
*flgJ*	flagellar rod assembly protein/muramidase	2.04	.020
Class 2 (middle) genes – *flhBAE* operon			
*flhB*	flagellar biosynthesis protein	1.67	.045
*flhA*	flagellar biosynthesis protein	2.30	.006
*flhE*	flagellar protein	4.27	.003
Class 2 (middle) genes – *fliAZY* operon			
*fliA*	Alternative sigma factor 28	1.02	NS
*fliZ*	RpoS antagonist	0.99	NS
*fliY*	Possible regulator of FliA	0.98	NS

Calculations of “fold change” are based on triplicate culture/microarray experiments for each treatment.

“NS” denotes “not significant” for p-values > 0.05.

**Table 5:** Downregulation of mRNA levels for “late” flagellar genes (flagellar filament, motor stator, and coregulated chemotaxis genes) in O157:H7 cells treated with 0.8% bile salts relative to control.

**Gene**	**Protein function**	**Fold change**	**p-value**
Class 2/Class 3 (middle & late) genes - *fliDST* operon			
*fliD*	flagellar capping protein	.47	< .001
*fliS*	flagellar protein, potentiates polymerization	.47	< .001
*fliT*	flagellar biosynthesis protein	.43	< .001
Class 2/Class 3 (middle & late) genes – *flgKL* operon			
*flgK*	flagellar hook-filament junction protein	.63	.024
*flgL*	flagellar hook-filament junction protein	.53	< .001
Class 3 (late) gene – *fliC*			
*fliC*	Flagellin	.53	< .001
Class 3 (late) genes – *tar-tap-cheZYBR* operon			
*tar*	methyl-accepting chemotaxis protein; sensor	.51	< .001
*tap*	chemotaxis for dipeptides & pyrimidines	.45	< .001
*cheR*	chemotaxis regulator, methyltransferase	.44	< .001
*cheB*	chemotaxis-specific methylesterase	.48	< .001
*cheY*	chemotaxis regulator, signaling to flagellar motor	.57	.001
*cheZ*	chemotaxis regulator, protein phosphatase for CheY	.55	< .001
Class 3 (late) genes – *motAB-cheAW* operon			
*motA*	stator, proton conductor component of flagella motor	.51	< .001
*motB*	stator, protein that enables flagellar motor rotation	.49	< .001
*cheA*	chemotaxis response regulator	.41	< .001
*cheW*	purine-binding chemotaxis protein	.45	< .001
Class 3 (late) genes -*tsr* & *aer*			
*tsr*	Methyl-accepting chemotaxis protein, serine sensor	.40	< .001
*aer*	aerotaxis sensor receptor, flavoprotein	.27	< .001

Calculations of “fold change” are based on triplicate culture/microarray experiments for each treatment.

To determine if the apparent transcriptional changes for flagellar and chemotaxis genes influenced cell motility, motility was analyzed using a soft agar motility assay. Cells cultured to log phase with and without 0.8% bile and then tested immediately on soft agar for motility showed no significant difference in the zone of swarming (data not shown). When 0.8% or 0.1% bile was incorporated into the agar medium for the motility assay, an increase in the diameter of the bacterial colonies was observed, suggesting an increase in swarming. However, incorporating bile into the soft agar destabilized the agar, rendering it less firm. Therefore, the change in motility may have been due to the differences in the viscosity of the agar with and without the addition of bile.

### Bile affects expression of genes involved in membrane permeability and bile efflux

A number of genes have been described as being involved in bacterial resistance to bile [1]. Bile resistance is accounted for by reduced influx of bile through changes in membrane permeability and by efflux pump mechanisms. Not surprisingly, in this study, mRNA levels for several of these genes were altered in *E. coli* O157:H7 cells treated with bile (Table 6). Among these genes, mRNA for one of the outer membrane porin genes, *ompF*, was reduced seven-fold compared to the untreated cells. Expression of the *ompC* porin appeared unchanged by the treatment. *ompF* expression is regulated by a network of factors. Several genes involved in regulation of *ompF* expression and bile efflux, including *micF*, *marA*, *marB*, *marR*, *acrA*, *acrB* and *acrR*, showed approximately two-fold increases in mRNA abundance following treatment with bile (Table 6). The regulatory gene *soxS* had three-fold reduced mRNA in the presence of bile when compared to the non-treated control.

**Table 6:** Changes in mRNA levels for genes related to membrane permeability and drug efflux in O157:H7 cells treated with 0.8% bile salts relative to control.

**Gene symbol**	**Gene name & function**	**Fold change**	**p-value**
*ompC*	Outer membrane protein C porin - permeability	0.97	NS
*ompF*	Outer membrane protein F porin - permeability	0.14	< .001
*micF*	Anti-sense RNA - negative regulation of OmpF translation	2.27	< .001
*marA*	Multiple antibiotic resistance (mar) protein A - transcription activator of mar regulon	2.01	< .001
*marB*	mar protein B	2.22	< .001
*marR*	mar operon repressor	1.80	.003
*soxS*	Regulatory protein SoxS - superoxide response regulon	0.37	< .001
*acrA*	Acriflavine resistance protein A - antibiotic and bile resistance	1.89	< .001
*acrB*	Acriflavine resistance protein B - antibiotic and bile resistance	2.21	< .001
*acrR*	Acriflavine resistance protein R –transcription regulator	2.83	< .001
*yceE*	Multidrug resistance protein - antibiotic and bile resistance	2.04	.002
*yojI*	Drug (microcin) efflux pump	2.49	< .001

Calculations of “fold change” are based on triplicate culture/microarray experiments for each treatment.

“NS” denotes “not significant” for p-values > 0.05.

We observed increased mRNA for additional genes likely involved in bile efflux. In addition to the *mar* and *acr* operons, the deoxycholate/multiple drug resistance gene *yceE* had a two-fold increased mRNA abundance due to bile treatment (Table 6). In addition, the drug efflux pump gene, *yojI*, also showed a 2.5-fold increased mRNA level for cells treated with bile.

### Bile affects genes related to adhesion

Bile-treated cells showed altered levels of mRNA for several genes implicated in adhesion and regulation of adhesion (Table 7). Bile treatment led to a three-fold reduction of mRNA for *tdcA*, a negative regulator of the outer membrane porin *ompA* gene, although *ompA* transcription was not significantly affected by bile. Related to the F9 fimbrial-adhesin operon, the *cadBA* locus was downregulated about six-fold by the addition of bile, while two genes of the F9 operon, *ydeQ* and *ydeR*, were increased 2.2-fold with bile treatment. The Z2200 gene encoding the major subunit of F9 fimbriae was only slightly affected by bile.

**Table 7:** Changes in mRNA levels for genes related to adhesion in O157:H7 cells treated with 0.8% bile salts relative to control.

**Gene symbol**	**Gene name & function**	**Fold change**	**p-value**
*ompA*	Outer membrane protein A porin - cell shape/integrity, adhesion	1.23	NS
*tdcA*	tdc operon transcription activator - negative regulation of ompA	0.30	< .001
*cadA*	lysine decarboxylase	0.17	< .001
*cadB*	cadaverine/lysine antiporter	0.17	< .001
*Z2200*	major F9 fimbrial subunit	1.40	.012
*ydeQ*	F9 fimbriae - predicted adhesin	2.23	.005
*ydeR*	F9 fimbriae - predicted adhesin	2.25	.027
*yodA*	Cadmium binding protein, implicated in adhesion	1.92	.004

Calculations of “fold change” are based on triplicate culture/microarray experiments for each treatment.

“NS” denotes “not significant” for p-values > 0.05.

### Bile influences mRNA of genes for iron scavenging and enhances growth of *E. coli O157:H7* during iron limiting conditions

*E. coli* O157:H7 cells growing in LB supplemented with bile salts showed increased mRNA levels for seventeen genes associated with iron acquisition and metabolism (Table 8). These genes include: *exbBD* and *tonB* encoding the Ton energy transduction system; *fhuACD* involved in ferrichrome uptake; *fhuF* encoding a siderophore-iron reductase; *feoABC* involved in ferrous iron uptake; *fepDGC* involved in ferric enterobactin transport; *entF* encoding an enterochelin synthase; *yheA* encoding bacterioferritin-associated ferredoxin; *yoeA* encoding a putative membrane receptor for an iron compound; and gene Z4383 encoding a putative iron compound permease. We checked whether extracellular iron concentrations might have changed during the course of bacterial growth. Before inoculation of bacteria, the iron concentration of LB, with or without bile salts added, was in the range of 1.4-1.5 mg/l. For logarithmic-phase cultures at which time cells were harvested for RNA extraction, iron concentration of the medium, with or without bile, was virtually unchanged at 1.3-1.4 mg/l.

**Table 8:** Iron acquisition genes upregulated in O157:H7 cells treated with 0.8% bile salts relative to control.

**Gene**	**Function**	**Fold increase**	**p-value**
*tonB*	Iron transport	2.76	< .001
*exbB*	TonB accessory protein	2.42	< .001
*exbD*	TonB accessory protein	2.85	< .001
*fhuA*	Ferrichrome (siderophore) uptake	5.40	< .001
*fhuC*	Ferrichrome (siderophore) uptake	2.70	< .001
*fhuD*	Ferrichrome (siderophore) uptake	1.76	.001
*fhuF*	Ferric iron reductase	2.44	< .001
*feoA*	Ferrous iron acquisition	2.88	< .001
*feoB*	Ferrous iron acquisition	2.57	< .001
*feoC*	DNA-binding, transcription regulator	2.81	< .001
*fepD*	Ferric enterobactin transport	4.18	< .001
*fepG*	Ferric enterobactin transport	2.78	< .001
*fepC*	ATP-binding component, ferric enterobactin transport	2.88	< .001
*entF*	Enterobactin (siderophore) synthesis	1.60	.006
*yheA*	Bacterioferritin-associated ferredoxin	1.84	.004
*yoeA*	Putative outer membrane receptor for iron compound (hemin) or colicin	1.95	.004
Z4383	Putative iron ABC transporter permease	1.61	.018

Calculations of “fold change” are based on triplicate culture/microarray experiments for each treatment.

**Figure 1:**
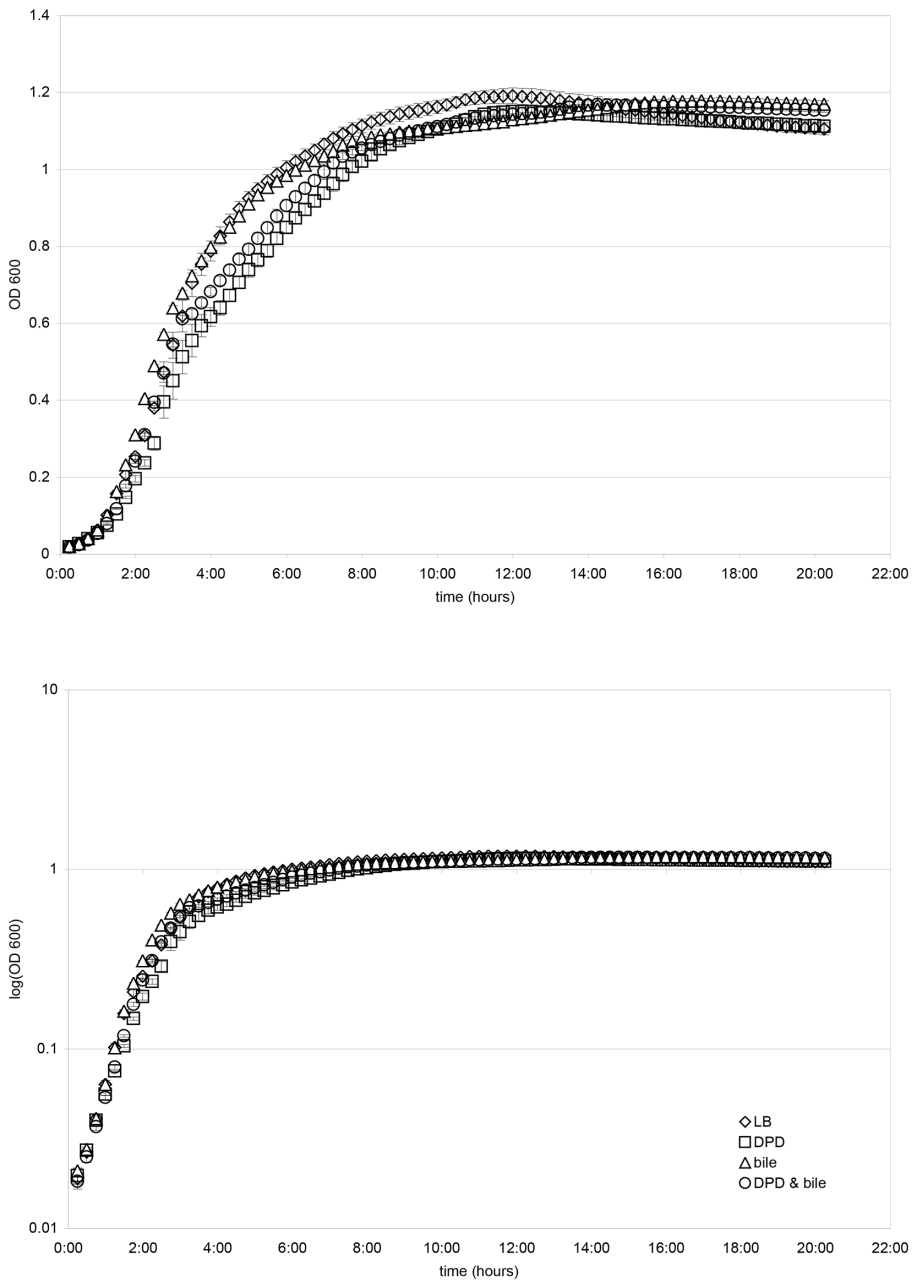
Growth of O157:H7 in LB medium, with and without 250 µM 2,2’-dipyridyl (DPD), with and without 0.8% bile salts. Four independent growth curve experiments were conducted and showed similar results. Linear and semi-log growth curve plots of OD_600_ over 20 hours for one of these experiments are presented in this figure. Error bars represent standard deviations for triplicate platings.

**Figure 2:**
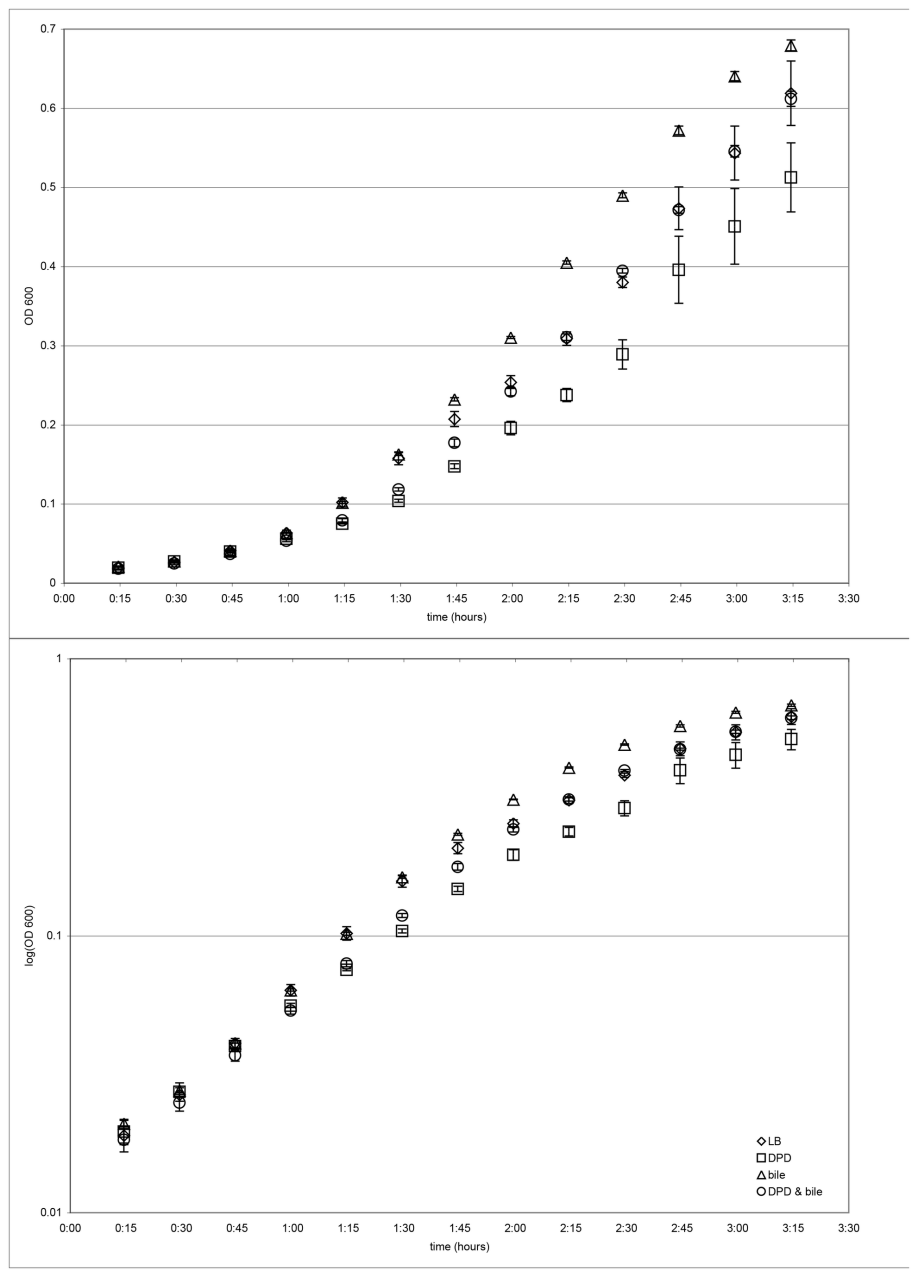
Effects of treatment with 250 µM 2,2’-dipyridyl (DPD) and/or 0.8% bile salts on exponential growth of O157:H7 in LB medium. Four independent growth curve experiments were conducted and showed similar results. Expanded linear and semi-log plots of OD_600_ for the first 3 hours 15 minutes of growth are provided for the same experimental data presented in Figure 1. Error bars represent standard deviations for triplicate platings.

While the total iron in the culture medium remained relatively unchanged following bile addition, bile may induce regulatory changes for iron scavenging. Alternatively, the available iron may be depleted by bile addition. Therefore, we determined the effect of addition of the iron chelating agent, 2,2’-dipyridyl (DPD) on growth of the bacteria in the presence and absence of bile. Growth of cells in LB broth, with and without bile salts, and with and without DPD, led to similar final cell yields (Figure 1). Growth curve plots obtained for four independent experiments indicated that cells growing with or without bile, and in the absence of DPD, grew best with average exponential growth phase doubling times of about 23-24 minutes (Figure 2, Table 9). Cells growing with DPD and without bile showed impaired growth in all four experiments, with an average exponential phase doubling time of about 32 minutes (Figure 2, Table 9). Cells treated with both bile and DPD showed less impairment in growth than did cells treated with DPD in the absence of bile, with an average exponential phase doubling time of about 27 minutes (Figure 2, Table 9). 

**Table 9:** Calculations of log growth phase doubling times for O157:H7 H7 grown in LB medium, with and without 250 µM 2,2’-dipyridyl (DPD), with and without 0.8% bile salts.

**Doubling times (in minutes**)** during exponential growth**				
**Experiment**	**LB only**	**with DPD**	**with bile**	**with DPD & bile**
1^st^	24.5	30.7	27.4	28.0
2^nd^	23.5	32.1	23.0	28.1
3^rd^	22.9	33.2	22.4	25.9
4^th^	23.2	31.9	23.0	26.7
Mean doubling time, four experiments (+/- standard error of mean)	23.5 (+/- 0.3)	32.0 (+/- 0.5)	24.0 (+/- 1.2)	27.2 (+/- 0.5)

Four independent growth curve experiments were conducted. Calculations of doubling times were made for the time interval 30 to 90 minutes (inclusive) when exponential growth curves (see Figure 2) were steepest.

## Discussion

### Bile effects on the LEE pathogenicity island

Bile treatment did not significantly alter the expression of Shiga-like toxin genes (Table 1). These data are in agreement with findings reported by Kus et al. [20] who have conducted a similar bile transcriptome experiment using the EDL933 strain of O157:H7.

Kus et al. [20] found no change in LEE mRNA levels between control and bile-treated O157-H7, whereas we found that most genes of the LEE pathogenicity island showed significantly reduced levels of mRNA in bile-treated O157:H7 cells (Table 2).

Several differences in protocol that may or may not account for the difference in bile’s effect on LEE gene expression seen between our study and the result reported by Kus et al. [20] include: the strain of O157:H7 used, the culture medium used to grow the bacteria, culture time, pH, and the concentration and type of bile salt mixture used. Kus et al. [20] observed changes after 1.5 hours of bile exposure, while our results were based on 3 hours of exposure. Kus et al. [20] reported subculturing cells into DMEM at pH 7.4 for their experiments; in our microarray experiments, the pH of LB medium was 7.0 before and after three hours of culture. Kus et al. [20] used a 0.15% concentration of a bile salt mixture (BSM, Sigma B3426) that may differ in composition from the bile salts (Difco 0129-02-4) used in our study. Of these differences in protocol, the major differences are likely to be the O157:H7 strain used, and the culture medium employed - growth in Dulbecco’s modified Eagle’s medium (DMEM) in the Kus experiment vs. growth in LB in our experiment.

Previously, O157:H7 and other related EHEC and EPEC bacteria grown in sodium bicarbonate-containing DMEM were reported to have altered protein expression profiles, including increased expression of LEE-encoded Esp protein [35], compared to when cells were grown in LB without bicarbonate. Abe et al. [36] reported that O157:H7 cells grown in DMEM attach more readily to Caco-2 (a human colorectal adenocarcinoma line) cells and express LEE-associated genes at higher levels than do O157:H7 cells pre-grown in LB. In analyzing the components of DMEM responsible for these properties, Abe et al. [36] found that sodium bicarbonate was the sole ingredient of DMEM responsible for the enhanced adherence and expression of the LEE genes. When increasing concentrations of sodium bicarbonate were included in the LB growth medium, transcription of LEE genes increased in a dose dependent manner. This study did not include testing of bile in conjunction with sodium bicarbonate for its effects on O157:H7 gene expression, however. Abe et al. [36] postulated that a relatively high level of sodium bicarbonate in the lower intestinal tract might act as an environmental signal promoting EHEC colonization of the large intestine.

A recent study by Yin et al. [37] indicates a role for bile both in inhibiting O157:H7 adherence to epithelial cells *in vitro* and in repressing expression of LEE-associated genes. Yin et al. [37] grew O157:H7 cells in MacConkey broth (MB), a medium lacking sodium bicarnbonate, both with and without bile salts. Bacteria grown in MB containing bile salts showed very little binding to monolayers of two different epithelial cell lines. O157:H7 cells pre-grown in MB without bile salts had increased adherence to the tissue culture cells. Yin et al. [37] also noted that O157:H7 cells pre-grown in brain heart infusion medium containing sodium bicarbonate (BHIN) without bile showed much greater adherence than did cells grown in MB lacking sodium bicarbonate. Addition of bile to BHIN slightly reduced bacterial adherence to the tissue culture cells and reduced expression of *eae*, *tir*, and *espD* by about 50%*.*

In summary, although the presence of sodium bicarbonate in various growth media appears to promote LEE gene expression and adherence by O157:H7 cells to varying degrees, addition of bile appears to have variable effects of LEE gene expression depending on the type of growth media used in these experiments. Although Kus et al. [20] noted no change in LEE expression for cells grown in DMEM (with sodium bicarbonate) with bile treatment, Yin et al. [37] noted a reduction in expression of LEE genes for cells grown in BHIN (with sodium bicarbonate) with bile similar to our findings obtained using LB medium without sodium bicarbonate.

With regard to the study described herein and to the study of Kus et al. [20], if protein levels of the LEE-encoded genes correlate with either these reduced or unchanged mRNA levels for the LEE genes, then the presence of bile in the small intestine may help protect against expression of LEE genes and the attaching-and-effacing phenotype by O157:H7 cells. Such protection might then diminish by the time O157:H7 cells reach the the large intestine where bile levels have been greatly reduced through active reabsorption in the ileum and where other factors may act to activate the LEE genes. Evidence suggesting that this scenario might actually occur is provided by the observations of Yin et al. [37]; their study noted that O157:H7 cells pre-grown in media containing bile salts, and consequently having reduced adherence and lowered expression of LEE genes *in vitro*, greatly increased expression of LEE genes and adhered quite well to host intestinal tissue after transfer into the ileal loops of infant pigs.

### Global effects of bile on the flagellar regulon

More than 50 genes make up the flagellar regulon responsible for flagellar assembly and chemotaxis [38]; regulation of this system is extremely complex [39]. Bile treatment had a global effect on the flagellar and chemotaxis regulon in *E. coli* O157:H7, with increased amounts of mRNA for the basal body genes, and reduced levels for the filament and chemotaxis-related genes. Initially, we thought it possible that bile-treated O157:H7 cells might produce fewer flagella or flagella with truncated filaments. This would be consistent with a study of a *cheY* (chemotaxis) mutant of *S* *. typhimurium* [40] that also displayed a reduction in expression of the late flagella and chemotaxis genes, as well as for genes of a Type III secretion system. The *S* *. typhimurium* mutant did not show increased mRNA for middle basal body-hook structure genes noted here for bile treated *E. coli* O157:H7. Colonies of the *S* *. typhimurium* mutant appeared less hydrated and mutant cells exhibited fewer and shorter flagella, with reduced swarming motility. The *Salmonella* study suggests a model whereby flagella sense certain features of the external environment, such as hydration or wetness, and this sensory input in turn modulates flagellar biosynthesis and other pathways [40]. While a different system was used here, a model involving cell hydration as for the *S* *. typhimurium* study may be relevant. Bile acts as a surfactant and may be an environmental cue for cell hydration. The cells may respond to this signal by modulation of flagella gene expression. In contrast to the *S* *. typhimurium* results, while changes in mRNA levels of flagella late genes were observed, no obvious difference in flagella length or numbers was observed here. Therefore, it is not yet clear how modulation of flagella gene expression by bile manifests as a physiological response.

Cells pretreated with bile salts and then plated on soft agar were as motile as non-treated cells. It may be that bile-treated cells plated on a soft agar plate without bile may quickly revert to a non-bile transcription expression profile, and so not reveal a difference in motility. Another motility assay test incorporating bile in the soft agar for the purpose of maintaining bile exposure during the motility assay proved problematic, since the presence of bile at a concentration as low as 0.1% destabilizes soft agar, rendering it less firm than agar medium without bile, thereby changing both the chemical and the physical environment of the cells.

In agreement with our data, Yin et al. [37] noted suppression of *fliC* transcription in O157:H7 cells grown *in vitro* due to the presence of bile salts; this study also detected virtually no transcription of *fliC* after O157:H7 bacteria were introduced into and grown in the ileal loops of pigs. Another related finding pertaining to bile and flagella expression is described by De Paepe et al. [41] who found that bile salts serve as a selective force in driving genetic diversification of commensal *E. coli* growing in the mouse gut; a K12 strain of *E. coli* grown either in the mouse gut or in chemostat culture with bile salts, gave rise to genetically divergent, non-motile subpopulations.

### Membrane permeability, efflux pumps and bile resistance

The ability to adjust the composition of structural components of the membrane in the face of changing and often hostile environmental conditions is crucial for bacterial survival. Alteration of membrane permeability and expression of efflux pumps are two important mechanisms allowing bacteria to resist antimicrobial agents such as antibiotics and detergents. Porins are a major class of outer membrane proteins that form channels or pores and allow non-specific nutrient intake and waste exchange [42]. OmpC and OmpF are the dominant porins expressed in *E. coli* and play a key role in regulating ion flux and outer membrane permeability. Transcription of *ompC* and *ompF* is affected by conditions such as osmolarity and temperature, and is controlled by a two-component signal transduction system comprised of EnvZ, a membrane sensor that monitors environmental conditions, and OmpR, a response regulator [43].

OmpF forms a slightly larger pore than OmpC, allowing bile salts to diffuse more readily through the larger OmpF channel [44,45]. Decreased expression of OmpF in *E. coli* occurs in response to bile salts and is associated with reduced influx of bile [46]. In the human intestine where bile is present, *ompF* is downregulated and OmpC becomes the predominant porin [42]. Bile-induced attenuation of OmpF synthesis involves post-transcriptional control by a porin regulatory system and several master regulatory proteins, including MarA, Rob, and SoxS [46,47]. The *marRAB* operon is associated with multiple antibiotic resistance as well as bile resistance. MarR is a repressor protein that binds to and silences the promoter for the *marRAB* operon. A variety of environmental agents such as salicylate can competitively bind to MarR, releasing it from the *mar* operator and activating transcription of the entire *marRAB* operon. The bile salt deoxycholate has been shown to interact with MarR and activate *marRAB* in *Salmonella* *typhimurium* [5].

MarA is a DNA-binding transcriptional activator involved with regulation of a large number of genes (i.e., a regulon) within the *E. coli* genome [47]. The other master regulators SoxS and Rob also cross-react with the MarA regulon. Increased synthesis of MarA or SoxS, or activation of constitutively-expressed Rob protein leads to increased synthesis of *micF* regulatory RNA. *micF* RNA in turn binds to *ompF* RNA, decreasing the synthesis of OmpF and reducing the influx of harmful agents such as bile. Increased synthesis and/or activation of these master regulators is also linked to increased expression of the multidrug efflux pump operon *acrAB*. The AcrAB efflux pump is able to export a number of antibiotics and antimicrobial agents, and plays a major role in efflux of bile salts [48].

YceE has also been implicated in multiple drug resistance and resistance to the bile salt deoxycholate in an *E. coli* K12 derivative lacking *acrAB* [49]. Accordingly, increased expression of *yceE* may contribute to bile-resistance in O157:H7. The YojI inner membrane protein [50] expels microcin, an antibacterial peptide produced by *E. coli*, and has not previously been associated with bile resistance. It remains to be determined whether YojI plays any role in bile efflux.

Our transcriptome data related to bile resistance is in agreement with the literature findings noted above, and indicate that a combination of decreased expression of OmpF and increased expression of the *acr* genes and *yceE* contribute to bile resistance of O157:H7 cells. Our findings are consistent with the results of Kus et al. [20] which also noted a decreased abundance of *ompF* mRNA and an increase in *acrAB* mRNA following 90 min of bile treatment.

O157:H7 cells treated with bile showed decreased mRNA for *soxS*, so SoxS is unlikely to contribute to *acrAB* activation. In *E. coli* K12 cells, bile salts have been shown to bind to and activate Rob, which in turn upregulates *arcAB* [51]. Both MarA and Rob may be important regulators of bile resistance in O157:H7.

Reduced expression of OmpF would limit permeability of not only bile salts, but also a variety of other molecules, including necessary nutrients. Coordinate upregulation of *ompC* would partially offset the restricted permeability caused by reduced OmpF levels, allowing passage of molecules smaller than bile salts. Alternately, a change in conformation to increase OmpC-mediated permeability would help to offset a decrease in OmpF expression. No change in *ompC* transcription level was noted in bile-treated O157:H7. We did note significantly decreased levels of mRNA for the *cadBA* operon in bile-treated O157:H7 cells. Downregulation of *cadA* may have some bearing on the dynamics of *ompF* downregulation and compensation by OmpC. *cadA* encodes the enzyme lysine decarboxylase that produces cadaverine as a by-product of lysine degradation. Cadaverine is normally associated with the outer membrane and has been shown to interact with OmpC and OmpF *in vitro*, inducing porin closure in a concentration-dependent manner [52]. Cadaverine may thus play a role in regulating membrane permeability. If decreased transcription of *cadA* corresponds to decreased levels of lysine decarboxylase activity, a bile-related decrease in cadaverine concentration may have consequences with regard to OmpC conformation. Reduced concentration of cadaverine may allow for an open instead of closed pore state for OmpC molecules. Increased OmpC-mediated permeability for small molecules could help offset reduced permeability associated with reduced OmpF expression.

Differences in amino acid composition of porins may also be important in altering permeability function in *E. coli*. In comparing wild-type vs. O157:H7 OmpC sequences, several amino acid changes are noted [53]. There are two amino acid differences between wild-type and O157:H7 OmpF, both occurring near the opening of the OmpF pore where they might alter pore size. There is also some evidence that porins may undergo post-translational modification to provide an additional level of regulation of permeability and membrane structure [53].

### Adhesion and cadBA

Adhesion to host tissue within the intestine is a crucial first step in colonization and infection for diarrheagenic *E. coli* [17,18]. Intimin is not the only adhesin mediating O157:H7 attachment to host tissue. An outer membrane protein, OmpA, important for maintaining membrane integrity and cell shape, has been identified as an adhesin for O157:H7 [18]. OmpA has been associated with the bacterial ability to bind to Caco-2 colorectal adenocarcinoma cells, HeLa adenocarcinoma cells, and plant surfaces [17,18]. Overexpression of OmpA in O157:H7 cells leads to a hyperadherent phenotype. Bacterial adhesion to epithelial cells and expression of OmpA may partially be under the negative control of *tdcA* [17], given that mutagenic inactivation of the *tdcA* gene causes both a hyperadherent phenotype and increased OmpA expression. *tdcA* codes for a transcriptional regulator of the *tdc* operon associated with transport and degradation of L-threonine. In our microarray analysis, bile-treated cells showed a decrease in *tdcA* mRNA, but insignificant change in *ompA* mRNA. Since bile treatment resulted in reduced mRNA for *eae*, encoding intimin, the adhesin important in later stage pathogenesis in the large intestine, and did not have a significant effect on *ompA*, other accessory adhesins may be important in early stage (pre-intimin) adhesion processes of O157:H7.

Several candidate accessory adhesins and regulatory mediators of adherence have been identified for O157:H7 [17,18]. Among these, CadA has been implicated as a negative regulator of EHEC adhesion to tissue culture cells and in colonization of the intestine in animal studies [19,54]. It has also been postulated that mutational inactivation or deletion of the *cadBA* locus in *Enterobacteriaceae* may be pathoadaptive, enhancing survival and pathogenic potential of bacteria within a host [55,56]. Downregulation of *cadBA* through bile exposure, as observed herein, may increase the pathogenic potential of O157:H7 cells once they enter the intestine where bile is present. Mutational disruption of the *cadA* locus has been shown to enhance adherence of EHEC to tissue culture cells [18] and to outcompete wild-type O157:H7 in colonization of rabbit intestine [19]. The *cadA*- mutation resulted in upregulation of flagella genes and the Z2200 gene encoding the major subunit of F9 fimbriae [19]. H7 flagella of O157:H7 have been shown to have adhesive properties, especially for mucins and bovine intestinal mucosa [14]. F9 fimbriae have been implicated in playing a role in O157:H7 colonization of cattle [16]. Our bile transcriptome data showed downregulation of the *cadBA* operon already noted, increased levels of mRNA for two genes of the F9 fimbrial-adhesin operon, *ydeQ* and *ydeR*, but only minor increase for the Z2200 gene previously described as encoding the major subunit of F9 [16].

### Bile salt activation of iron acquisition genes

Iron is the only nutrient not freely available to bacteria in the human host [57]. Iron acquisition is necessary for bacterial survival, and accordingly, can be said to play an important role in bacterial pathogenesis. Inside the host, bacteria can acquire iron through production of accessory toxins, hemolysins and ferric ion chelators. Control of gene expression in response to low iron availability is an important feature of bacterial growth and can involve the expression of several genes involved in virulence. With regard to bacterial growth, the human intestine is considered to be an iron-restricted environment. Iron is actively absorbed by enterocytes in the duodenum and is sequestered by lactoferrin present in bile secretion and the intestinal mucosal layer.

In our experiments using a rich growth medium, iron is plentiful for supporting growth of bacteria through logarithmic growth phase. Surprisingly, O157:H7 cells treated with bile salts showed increased mRNA for seventeen genes related to iron acquisition. Such changes would be expected to occur in response to low iron conditions. Measurements of iron concentration in the growth medium showed almost no change during the course of cell growth through log phase and cell harvesting for RNA extraction. Bile activation of iron acquisition genes in O157:H7 cells growing in the presence of abundant iron raised the question of whether this response to bile signal might affect cell growth and provide some growth advantage in an iron-depleted environment. To address this question, cell growth was examined in the presence and absence of both bile salts and the iron-chelating agent DPD. Inclusion of both bile salts and DPD in the growth medium partially mimics the changing conditions of the duodenum where bile is introduced and an iron-limited environment is being created. The results presented in Figure 2 suggest that DPD treatment has a significant effect in reducing O157:H7 growth rate, and that bile partially counteracts this deleterious effect of iron depletion on O157:H7 growth. During the three hours or so that it takes for digested food (with bacteria) to transit through the human small intestine, bile induction of iron acquisition genes in O157:H7 may have a protective or beneficial effect on O157:H7 growth in the small intestine. In our experiments, exposure to bile salts partially relieves the iron stress response of slowed growth of O157:H7 caused by DPD treatment. Apparently, O157:H7 bacteria respond to bile salts in a manner that enables the bacteria to more successfully counteract the stress of iron chelation caused by DPD.

It is intriguing that bile salt treatment by itself triggers expression of an iron stress response for O157:H7 cells growing in iron rich medium. In the host, bacteria are first exposed to bile in the duodenum, the same site where an iron-scarce environment is being created. It appears that for an intestinal pathogen such as O157:H7, the genetic response to bile has been associated through evolution and repeated passage in and out of mammalian hosts with a reduced availability of iron associated with the small intestine. Such a cross-linking of genetic and biochemical pathways in response to entering a host environment from an external setting has been recently described for *E. coli* and its response to changes in temperature and oxygen levels associated with entry into the gastrointestinal tract [58].

Bacteria encountering signals indicative of the intestinal environment, in the case of our experiment, the presence of bile in the growth media, may well be at a selective advantage if they can immediately activate related genetic and biochemical pathways (e.g., iron acquisition) required for survival in the intestine. Exposure of O157:H7 to bile appears to effectively be a signal to the bacteria that they have entered the small intestine and must prepare to deal with low iron availability.

In conclusion, during the course of infection within the human host, O157:H7 must survive a variety of host defense mechanisms, including exposure to the antimicrobial agent bile during passage through the small intestine. The findings presented here indicate that O157:H7 responds to bile through wide-ranging changes in gene transcription. Our study may provide insight into how this pathogen adapts to the changing environmental conditions and host defenses encountered during human infection.

## References

[1] Begley M, Gahan CGM, Hill C (2005) The interaction between bacteria and bile. FEMS Microbiol Rev 29: 625-651. doi:10.1016/j.femsre.2004.09.003. PubMed: 16102595. 16102595 10.1016/j.femsre.2004.09.003

[2] Kandell RL, Bernstein C (1991) Bile salt induction of DNA damage in bacterial cells: implications for colon cancer. Nutr Cancer 16: 227-238. doi:10.1080/01635589109514161. PubMed: 1775385. 1775385 10.1080/01635589109514161

[3] Bernstein C, Bernstein H, Payne CM, Beard SE, Schneider J (1999) Bile salt activation of stress response promoters in *Escherichia* *coli* . Curr Microbiol 39: 68-72. doi:10.1007/s002849900420. PubMed: 10398829. 10398829 10.1007/s002849900420

[4] Gunn JS (2000) Mechanisms of bacterial resistance and response to bile. Microbes Infect 2: 907-913. doi:10.1016/S1286-4579(00)00392-0. PubMed: 10962274. 10962274 10.1016/s1286-4579(00)00392-0

[5] Prouty AM, Brodsky IE, Falkow S, Gunn JS (2004) Bile-salt-mediated induction of antimicrobial and bile resistance in *Salmonella* *typhimurium* . Microbiology 150: 775-783. doi:10.1099/mic.0.26769-0. PubMed: 15073288. 15073288 10.1099/mic.0.26769-0

[6] Nataro JP, Kaper JB (1998) Diarrheagenic *Escherichia* *coli* . Clin Microbiol Rev 11: 142-201. PubMed: 9457432. 9457432 10.1128/cmr.11.1.142 PMC121379

[7] Paton JC, Paton AW (1998) Pathogenesis and diagnosis of Shiga toxin-producing *Escherichia* *coli* infections. Clin Microbiol Rev 11: 450-479. PubMed: 9665978. 9665978 10.1128/cmr.11.3.450 PMC88891

[8] Donnenberg MS, Whittam TS (2001) Pathogenesis and evolution of virulence in enteropathogenic and enterohemorrhagic Escherichia coli. J Clin Invest 107: 539-548. doi:10.1172/JCI12404. PubMed: 11238553. 11238553 10.1172/JCI12404 PMC199431

[9] Garmendia J, Frankel G, Crepin VF (2005) Enteropathogenic and enterohemorrhagic Escherichia coli infections: translocation, translocation, translocation. Infect Immun 73: 2573-2585. doi:10.1128/IAI.73.5.2573-2585.2005. PubMed: 15845459. 15845459 10.1128/IAI.73.5.2573-2585.2005 PMC1087358

[10] Abu-Ali GS, Ouellette LM, Henderson ST, Whittam TS, Manning SD (2010) Differences in adherence and virulence gene expression between two outbreak strains of enterohemorrhagic Escherichia coli O157:H7. Microbiology 156: 408-419. 19892762 10.1099/mic.0.033126-0 PMC2890088

[11] Abu-Ali GS, Ouellette LM, Henderson ST, Lacher DW, Riordan JT et al. (2010) Increased adherence and expression of virulence genes in a lineage of *Escherichia* *coli* O157:H7 commonly associated with human infections. PLOS ONE 5: e10167. doi:10.1371/journal.pone.0010167. PubMed: 20422047. 20422047 10.1371/journal.pone.0010167 PMC2858043

[12] Kulasekara BR, Jacobs M, Zhou Y, Wu Z, Sims E et al. (2009) Analysis of the genome of the *Escherichia* *coli* O157:H7 2006 spinach-associated outbreak isolate indicates candidate genes that may enhance virulence. Infect Immun 77: 3713-3721. doi:10.1128/IAI.00198-09. PubMed: 19564389. 19564389 10.1128/IAI.00198-09 PMC2738036

[13] Manning SD, Motiwala AS, Springman AC, Qi W, Lacher DW et al. (2008) Variation in virulence among clades of *Escherichia* *coli* O157:H7 associated with disease outbreaks. Proc Natl Acad Sci U S A 105: 4868-4873. doi:10.1073/pnas.0710834105. PubMed: 18332430. 18332430 10.1073/pnas.0710834105 PMC2290780

[14] Erdem AL, Avelino F, Xicohtencatl-Cortes J, Girón JA (2007) Host protein binding and adhesive properties of H6 and H7 flagella of attaching and effacing *Escherichia* *coli* . J Bacteriol 189: 7426-7435. doi:10.1128/JB.00464-07. PubMed: 17693516. 17693516 10.1128/JB.00464-07 PMC2168434

[15] Ho TD, Davis BM, Ritchie JM, Waldor MK (2008) Type 2 secretion promotes enterohemorrhagic Escherichia coli adherence and intestinal colonization. Infect Immun 76: 1858-1865. doi:10.1128/IAI.01688-07. PubMed: 18316380. 18316380 10.1128/IAI.01688-07 PMC2346697

[16] Low AS, Dziva F, Torres AG, Martinez JL, Rosser T et al. (2006) Cloning, expression, and characterization of fimbrial operon F9 from enterohemorrhagic *Escherichia* *coli* O157:H7. Infect Immun 74: 2233-2244. 16552054 10.1128/IAI.74.4.2233-2244.2006 PMC1418889

[17] Torres AG, Jeter C, Langley W, Matthysse AG (2005) Differential binding of *Escherichia* *coli* O157:H7 to alfalfa, human epithelial cells, and plastic is mediated by a variety of surface structures. Appl Environ Microbiol 71: 8008-8015. doi:10.1128/AEM.71.12.8008-8015.2005. PubMed: 16332780. 16332780 10.1128/AEM.71.12.8008-8015.2005 PMC1317338

[18] Torres AG, Kaper JB (2003) Multiple elements controlling adherence of enterohemorrhagic Escherichia coli O157:H7 to HeLa cells. Infect Immun 71: 4985-4995. doi:10.1128/IAI.71.9.4985-4995.2003. PubMed: 12933841. 12933841 10.1128/IAI.71.9.4985-4995.2003 PMC187298

[19] Vazquez-Juarez RC, Kuriakose JA, Rasko DA, Ritchie JM, Kendall MM et al. (2008) CadA negatively regulates Escherichia coli O157:H7 adherence and intestinal colonization. Infect Immun 76: 5072-5081. 18794292 10.1128/IAI.00677-08 PMC2573373

[20] Kus JV, Gebremedhin A, Dang V, Tran S-L, Serbanescu A, Foster DB (2011) Bile salts induce resistance to polymyxin in enterohemorrhagic Escherichia coli O157:H7. J Bacteriol 193: 4509-4515. 21725004 10.1128/JB.00200-11 PMC3165498

[21] Pyle BH, Broadaway SC, McFeters GA (1999) Sensitive detection of *Escherichia* *coli* O157:H7 in food and water by immunomagnetic separation and solid-phase laser cytometry. Appl Environ Microbiol 65: 1966-1972. PubMed: 10223987. 10223987 10.1128/aem.65.5.1966-1972.1999 PMC91284

[22] McGraw EA, Li J, Selander RK, Whittam TS (1999) Molecular evolution and mosaic structure of alpha, beta, and gamma intimins of pathogenic *Escherichia* *coli* . Mol Biol Evol 16: 12-22. doi:10.1093/oxfordjournals.molbev.a026032. PubMed: 10331248. 10331248 10.1093/oxfordjournals.molbev.a026032

[23] Reid SD, Selander RK, Whittam TS (1999) Sequence diversity of flagellin (fliC) alleles in pathogenic *Escherichia* *coli* . J Bacteriol 181: 153-160. PubMed: 9864325. 9864325 10.1128/jb.181.1.153-160.1999 PMC103544

[24] Blazejczyk M, Miron M, Nadon R (2007) FlexArray: A statistical data analysis software for gene expression microarrays. Genome Quebec, Montreal, Canada Available: http://genomequebec.mcgill.ca/FlexArray.

[25] Wu Z, Irizarry RA, Gentleman R, Murillo FM, Spencer F (2004) A Model Based Background Adjustment for Oligonucleotide Expression Arrays. Working Papers, Department of Biostatistics, Johns Hopkins University.

[26] Baldi P, Long AD (2001) A Bayesian framework for the analysis of microarray expression data: regularized t-test and statistical inferences of gene changes. Bioinformatics 17: 509-519. Available: http://cybert.microarray.ics.uci.edu/. doi:10.1093/bioinformatics/17.6.509. PubMed: 11395427. 11395427 10.1093/bioinformatics/17.6.509

[27] Sturn A, Quackenbush J, Trajanoski Z (2002) Genesis: cluster analysis of microarray data. Bioinformatics 18: 207-208. Available: http://genome.tugraz.at/genesisclient/genesisclient_description.shtml. doi:10.1093/bioinformatics/18.1.207. PubMed: 11836235. 11836235 10.1093/bioinformatics/18.1.207

[28] Rozen S, Skaletsky HJ (2000) Primer3 on the WWW for general users and for biologist programmers. In Krawetz S Misener S Bioinformatics Methods and Protocols: Methods in Molecular Biology. Totowa, NJ: Humana Press pp. 365-386. Available: http://sourceforge.net/projects/primer3/. 10.1385/1-59259-192-2:365 10547847

[29] Viveiros M, Dupont M, Rodrigues L, Couto I, Davin-Regli A et al. (2007) Antibiotic stress, genetic response and altered permeability of *E.* *coli* . PLOS ONE 2: e365. doi:10.1371/journal.pone.0000365. PubMed: 17426813. 17426813 10.1371/journal.pone.0000365 PMC1838523

[30] Degen LP, Phillips SF (1996) Variability of gastrointestinal transit in healthy women and men. Gut 39: 299-305. doi:10.1136/gut.39.2.299. PubMed: 8977347. 8977347 10.1136/gut.39.2.299 PMC1383315

[31] Levitt MD, Bond JH (1980) Flatulence. Annu Rev Med 31: 127-137. doi:10.1146/annurev.me.31.020180.001015. PubMed: 6772089. 6772089 10.1146/annurev.me.31.020180.001015

[32] He G, Shankar RA, Chzhan M, Samouilov A, Kuppusamy P et al. (1999) Noninvasive measurement of anatomic structure and intraluminal oxygenation in the gastrointestinal tract of living mice with spatial and spectral EPR imaging. Proc Natl Acad Sci U S A 96: 4586-4591. doi:10.1073/pnas.96.8.4586. PubMed: 10200306. 10200306 10.1073/pnas.96.8.4586 PMC16376

[33] Wiles S, Pickard KM, Peng K, MacDonald TT, Frankel G (2006) *In* *vivo* bioluminescence imaging of the murine pathogen *Citrobacter* *rodentium* . Infect Immun 74: 5391-5396. doi:10.1128/IAI.00848-06. PubMed: 16926434. 16926434 10.1128/IAI.00848-06 PMC1594854

[34] Jones SA, Chowdhury FZ, Fabich AJ, Anderson A, Schreiner DM et al. (2007) Respiration of *Escherichia* *coli* in the mouse intestine. Infect Immun 75: 4891-4899. doi:10.1128/IAI.00484-07. PubMed: 17698572. 17698572 10.1128/IAI.00484-07 PMC2044527

[35] Ebel F, Deibel C, Kresse AU, Guzmán CA, Chakraborty T (1996) Temperature- and medium-dependent secretion of proteins by Shiga toxin-producing *Escherichia* *coli* . Infect Immun 64: 4472-4479. PubMed: 8890194. 8890194 10.1128/iai.64.11.4472-4479.1996 PMC174400

[36] Abe H, Tatsuno I, Tobe T, Okutani A, Sasakawa C (2002) Bicarbonate ion stimulates the expression of locus of enterocype effacement-encoded genes in enterohemorrhagic Escherichia coli O157:H7. Infect Immun 70: 3500-3509. 12065489 10.1128/IAI.70.7.3500-3509.2002 PMC128104

[37] Yin X, Zhu J, Feng Y, Chambers JR, Gong J, Gyles CL (2011) Differential gene expression and adherence of Escherichia coli O157:H7 in vitro and in ligated pig intestines. PLOS ONE 6: e17424. doi:10.1371/journal.pone.0017424. PubMed: 21387009. 21387009 10.1371/journal.pone.0017424 PMC3046156

[38] Chilcott GS, Hughes KT (2000) Coupling of flagellar gene expression to flagellar assembly in *Salmonella* *enterica* serovar Typhimurium and *Escherichia* *coli* . Microbiol Mol Biol Rev 64: 694-708. doi:10.1128/MMBR.64.4.694-708.2000. PubMed: 11104815. 11104815 10.1128/mmbr.64.4.694-708.2000 PMC99010

[39] Kalir S, McClure J, Pabbaraju K, Southward C, Ronen M et al. (2001) Ordering genes in a flagella pathway by analysis of expression kinetics from living bacteria. Science 292: 2080-2083. doi:10.1126/science.1058758. PubMed: 11408658. 11408658 10.1126/science.1058758

[40] Wang Q, Suzuki A, Mariconda S, Porwollik S, Harshey RM (2005) Sensing wetness: a new role for the bacterial flagellum. EMBO J 24: 2034-2042. doi:10.1038/sj.emboj.7600668. PubMed: 15889148. 15889148 10.1038/sj.emboj.7600668 PMC1142604

[41] De Paepe M, Gaboriau-Routhiau V, Rainteau D, Rakotobe S, Taddei F et al. (2011) Trade-off between bile resistance and nutritional competence drives *Escherichia* *coli* diversification in the mouse gut. PLOS Genet 7: e1002107. doi:10.1371/journal.pgen.1002107. PubMed: 21698140. 21698140 10.1371/journal.pgen.1002107 PMC3116916

[42] Nikaido H (2003) Molecular basis of bacterial outer membrane permeability revisited. Microbiol Mol Biol Rev 67: 593-656. doi:10.1128/MMBR.67.4.593-656.2003. PubMed: 14665678. 14665678 10.1128/MMBR.67.4.593-656.2003 PMC309051

[43] Pratt LA, Hsing W, Gibson KE, Silhavy TJ (1996) From acids to *osmZ*: multiple factors influence synthesis of OmpF and OmpC. Mol Microbiol 20: 911-917. doi:10.1111/j.1365-2958.1996.tb02532.x. PubMed: 8809744. 8809744 10.1111/j.1365-2958.1996.tb02532.x

[44] Nikaido H, Rosenberg EY (1983) Porin channels in *Escherichia* *coli*: studies with liopsomes reconstituted from purified proteins. J Bacteriol 153: 241-252. PubMed: 6294049. 6294049 10.1128/jb.153.1.241-252.1983 PMC217362

[45] Nikaido H, Rosenberg EY, Foulds J (1983) Porin channels in *Escherichia* *coli*: studies with beta-lactams in intact cells. J Bacteriol 153: 232-240. PubMed: 6294048. 6294048 10.1128/jb.153.1.232-240.1983 PMC217361

[46] Alekshun MN, Levy SB (1997) Regulation of chromosomally mediated multiple antibiotic resistance: the *mar* regulon. Antimicrob Agents Chemother 41: 2067-2075. PubMed: 9333027. 9333027 10.1128/aac.41.10.2067 PMC164072

[47] Barbosa TM, Levy SB (2000) Differential expression of over 60 chromosomal genes in *Escherichia* *coli* by constitutive expression of MarA. J Bacteriol 182: 3467-3474. doi:10.1128/JB.182.12.3467-3474.2000. PubMed: 10852879. 10852879 10.1128/jb.182.12.3467-3474.2000 PMC101932

[48] Thanassi DG, Cheng LW, Nikaido H (1997) Active efflux of bile salts by *Escherichia* *coli* . J Bacteriol 179: 2512-2518. PubMed: 9098046. 9098046 10.1128/jb.179.8.2512-2518.1997 PMC178997

[49] Nishino K, Yamaguchi A (2001) Analysis of a complete library of putative drug transporter genes in *Escherichia* *coli* . J Bacteriol 183: 5803-5812. doi:10.1128/JB.183.20.5803-5812.2001. PubMed: 11566977. 11566977 10.1128/JB.183.20.5803-5812.2001 PMC99656

[50] Pomares MF, Vincent PA, Farías RN, Salomón RA (2008) Protective action of ppGpp in microcin J25-sensitive strains. J Bacteriol 190: 4328-4234. doi:10.1128/JB.00183-08. PubMed: 18408024. 18408024 10.1128/JB.00183-08 PMC2446768

[51] Rosenberg EY, Bertenthal D, Nilles ML, Bertrand KP, Nikaido H (2003) Bile salts and fatty acids induce the expression of *Escherichia* *coli* AcrAB multidrug efflux pump through their interaction with Rob regulatory protein. Mol Microbiol 48: 1609-1619. doi:10.1046/j.1365-2958.2003.03531.x. PubMed: 12791142. 12791142 10.1046/j.1365-2958.2003.03531.x

[52] delaVega AL, Delcour AH (1995) Cadaverine induces closing of *E.* *coli* porins. EMBO J 14: 6058-6065. PubMed: 8846798. 8846798 10.1002/j.1460-2075.1995.tb00294.x PMC394726

[53] Martinez MB, Flickinger M, Higgins LA, Krick T, Nelsestuen GL (2001) Reduced outer membrane permeability of *Escherichia* *coli* O157:H7: suggested role of modified outer membrane porins and theoretical function in resistance to antimicrobial agents. Biochemistry 40: 11965-11974. doi:10.1021/bi0109515. PubMed: 11580272. 11580272 10.1021/bi0109515

[54] Torres AG, Vazquez-Juarez RC, Tutt CB, Garcia-Gallegos JG (2005) Pathoadaptive mutation that mediates adherente of Shiga toxin-producing *Escherichia* *coli* O111. Infect Immun 73: 4766-4776. doi:10.1128/IAI.73.8.4766-4776.2005. PubMed: 16040989. 16040989 10.1128/IAI.73.8.4766-4776.2005 PMC1201210

[55] Maurelli AT (2007) Black holes, antivirulence genes, and gene inactivation in the evolution of bacterial pathogens. FEMS Microbiol Lett 267: 1-8. PubMed: 17233672. 17233672 10.1111/j.1574-6968.2006.00526.x

[56] Torres AG (2009) The *cad* locus of *Enterobacteriaceae*: more than just lysine decarboxylation. Anaerobe 15: 1-6. doi:10.1016/j.anaerobe.2008.05.002. PubMed: 18572426. 18572426 10.1016/j.anaerobe.2008.05.002

[57] Ratledge C, Dover LG (2000) Iron metabolism in pathogenic bacteria. Annu Rev Microbiol 54: 881-941. doi:10.1146/annurev.micro.54.1.881. PubMed: 11018148. 11018148 10.1146/annurev.micro.54.1.881

[58] Tagkopoulos I, Liu Y-C, Tavazoie S (2008) Predictive behavior within microbial genetic networks. Science 320: 1313-1317. doi:10.1126/science.1154456. PubMed: 18467556. 18467556 10.1126/science.1154456 PMC2931280

